# Leveraging Interdisciplinary Education Toward Securing the Future of Connected Health Research in Europe: Qualitative Study

**DOI:** 10.2196/14020

**Published:** 2019-11-13

**Authors:** Ioanna Chouvarda, Nicola Mountford, Vladimir Trajkovik, Tatjana Loncar-Turukalo, Tara Cusack

**Affiliations:** 1 Lab of Computing, Medical Informatics & Biomedical Imaging Technologies School of Medicine Aristotle University of Thessaloniki Thessaloniki Greece; 2 School of Business Maynooth University Maynooth, County Kildare Ireland; 3 Faculty of Computer Science and Engineering Saints Cyril and Methodius University Skopje the Former Yugoslav Republic of Macedonia; 4 Faculty of Technical Sciences University of Novi Sad Novi Sad Serbia; 5 Health Sciences Centre School of Public Health, Physiotherapy and Sports Science University College Dublin Dublin Ireland

**Keywords:** connected health, interdisciplinary studies, interdisciplinary research, problem-based learning, technology and health

## Abstract

**Background:**

Connected health (CH) technologies have resulted in a paradigm shift, moving health care steadily toward a more patient-centered delivery approach. CH requires a broad range of disciplinary expertise from across the spectrum to work in a cohesive and productive way. Building this interdisciplinary relationship at an earlier stage of career development may nurture and accelerate the CH developments and innovations required for future health care.

**Objective:**

This study aimed to explore the perceptions of interdisciplinary CH researchers regarding the design and delivery of an interdisciplinary education (IDE) module for disciplines currently engaged in CH research (engineers, computer scientists, health care practitioners, and policy makers). This study also investigated whether this module should be delivered as a taught component of an undergraduate, master’s, or doctoral program to facilitate the development of interdisciplinary learning.

**Methods:**

A qualitative, cross-institutional, multistage research approach was adopted, which involved a background study of fundamental concepts, individual interviews with CH researchers in Greece (n=9), and two structured group feedback sessions with CH researchers in Ireland (n=10/16). Thematic analysis was used to identify the themes emerging from the interviews and structured group feedback sessions.

**Results:**

A total of two sets of findings emerged from the data. In the first instance, challenges to interdisciplinary work were identified, including communication challenges, divergent awareness of state-of-the-art CH technologies across disciplines, and cultural resistance to interdisciplinarity. The second set of findings were related to the design for interdisciplinarity. In this regard, the need to link research and education with real-world practice emerged as a key design concern. Positioning within the program context was also considered to be important with a need to balance early intervention to embed integration with later repeat interventions that maximize opportunities to share skills and experiences.

**Conclusions:**

The authors raise and address challenges to interdisciplinary program design for CH based on an abductive approach combining interdisciplinary and interprofessional education literature and the collection of qualitative data. This recipe approach for interdisciplinary design offers guidelines for policy makers, educators, and innovators in the CH space. Gaining insight from CH researchers regarding the development of an IDE module has offered the designers a novel insight regarding the curriculum, timing, delivery, and potential challenges that may be encountered.

## Introduction

### Background

The population of Europe is aging. Life expectancy is estimated to increase by more than a full year between 2016 and 2021—from 73 to 74.1 years—bringing the number of people aged over 65 years to more than 656 million or 11.5% of the total population [1]. Coupled with the increased incidence of chronic disease, this illustrates the challenges being faced by the health care system. Currently, hospital admissions consume more than 37 million bed days each year across the European Union (EU). However, the digital transformation of health and care, a key component of the EU’s Digital Single Market, offers tremendous potential for improving the prevention, detection, and management of chronic diseases as well as improving health system management and research [2]. New and innovative ways to maintain and enhance health are required. Connected health (CH) describes the use of technology to provide health care services in a more flexible and cost-effective way for both citizens and health care practitioners. CH refers to [3]:

A conceptual model for health management where devices, services or interventions are designed around the person’s needs, and health-related data is shared, in such a way that the person can receive care in the most proactive and efficient manner possible. All stakeholders in the process are “connected” by means of timely sharing and presentation of accurate and pertinent information regarding patient status through smarter use of data, devices, communication platforms and people.

CH can be used by both clinicians and citizens to enable better and more efficient use of scarce health care resources. It promises a paradigm shift with novel technologies being used to create and develop links between individuals and communities, health and disease, and different health actors (eg, citizens, patients, clinicians, and policy makers), thereby enabling citizens and health care practitioners to make better decisions about health care.

CH interventions such as home-based exercise programs can contribute to CH impact via improvement in patient adherence [4], but they require reshaping of health care. Therefore, motivating stakeholder involvement to embrace such change requires some existing evidence on CH effectiveness and advantages. Unfortunately, there is still some distance to go in terms of proving the effectiveness of such programs [5]. The challenges in achieving such evidence-based proof are both technological and medical. The role of digital health education and the resulting literacy of CH-involved stakeholders have been highlighted [6], especially with regard to quality and safety concerns in health data as well as the fear of unintended consequences of technology use. A comprehensive set of evidence-based guidelines for CH might allow it to achieve its full potential [7]. Working toward the necessary evidence and medical guidelines will, however, require the development of a new CH culture that is actively embraced by CH stakeholders across multiple domains and disciplines.

Adopting a CH approach to health care could enhance the delivery of a more integrated health care system [8]. For health care to be *integrated*, it must connect “inputs, delivery, management and organization of services related to diagnosis, treatment, care, rehabilitation and health promotion” [9]. Such coordination of care requires, however, a corresponding coordination of multiple professional camps, such as clinicians, medical device engineers, health care managers, and policy makers. CH can offer a key building block in this regard, offering the tools to coordinate consistent care across settings and over time and bringing together the various groups involved in patient care to decide, organize, and deliver services [8].

Concepts central to both the connectedness and integration aspects of the CH approach are sharing of information, involvement and collaboration of multiple professions, redesign of care models, temporal and organizational continuum, new care and business models, and leveraging of services via technology. The development of CH technologies, therefore, requires that professionals from across both the health care and technology disciplines work together in a more integrated and cohesive way. This implies a good understanding of the skills each discipline brings to the team and the health needs they intend to address [9]. Furthermore, these new services and care models involve innovation, which, in turn, involves teamwork and synthesis, including also a closer integration between health and biomedical sciences, as well as information and communications technology (ICT) sciences [10]. A natural driver for such developments is education. Some work has already been undertaken in this area, and the European Network for the Joint Evaluation of Connected Health Technologies (ENJECT) report [11] refers to education programs across Europe and the extent to which they are creating the conditions and skills necessary for the widespread adoption of CH. It is essential to educate the next generation of researchers, clinicians, scientists, and decision makers about the importance of CH so that they, in turn, will be able to empower citizens to engage with CH as fully as possible through their own professional lives.

Medical students are envisioned as the frontline of *digital natives*, and telemedicine education is proposed for their preclinical and clinical curricula [12]. Recognizing that telemedicine and telehealth will play an increasingly essential role in the delivery of health care, the American Medical Association has called for telehealth to become a core competency of medical students. They do so in the knowledge that studies have demonstrated that a principal reason why physicians do not practice telehealth is lack of education despite patients’ interest in telehealth [10].

Slovensky et al [13] proposed a set of mobile health (mHealth) skills for health professionals as a necessary enhancement for clinical training programs that ultimately would benefit both providers and patients. These included (1) digital communication skills, (2) technology literacy and usage skills, (3) mHealth products and services, (4) regulatory and compliance issues, and (5) the technology business case. In the CH context, two key areas of education have been identified for future nursing graduates: (1) the actual use of new technologies and (2) managing and making sense of the data produced (data literacy to interpret and make use of ambient data) [14]. These are clear indicators of the need to consider CH education in different health care professions.

However, CH involves a broad spectrum of disciplines extending beyond those directly associated with traditional health care and health care delivery. Recent studies and research programs have begun to recognize that engineers, computer scientists, information technology designers, and policy makers must together engage in interdisciplinary research to deliver on the promise of CH. Xu et al [10] emphasize the need for the discovery of new diagnostic tools and treatments by using a multidisciplinary and highly collaborative approach. Mountford et al [15] extend this to the education of CH researchers, presenting the need for comprehensive training and research program by embracing all key elements—technical, social, and economic sciences—required to produce researchers and project outcomes that are capable of meeting existing and future needs in cancer rehabilitation. We argue that relevant professionals outside of the health care field (beyond junior researchers) also need to understand the nuances and requirements of CH within the domain of modern medicine. For all disciplines to participate in the development of CH and to ensure that innovations are fit for purpose, it is imperative that all disciplines work together to enable a more informed and potentially creative future. It is, therefore, apparent that CH interdisciplinary team working needs to be established both in day-to-day practice and in the education programs that prepare professionals for such practice.

### The Research Question

The main premise underpinning this research is that modeling interdisciplinary team working behaviors during the education process is a prerequisite for productive interdisciplinary cooperation in CH research and development as well as practice. Clear understanding of benefits, potential, and relevance of interdisciplinary collaboration must be learned and practiced to yield its full potential in the field. Education of health care professionals should embrace the technological innovation that is empowering the field of medicine today. Engineering curricula, being far more flexible in continuous adaptations to the exponential progress in the field, have already encompassed enabling technology for health. However, only bringing the interdisciplinary teamwork into the classroom will ensure broader acceptance and understanding of the fundamental CH concepts. Investment at the education level will eventually facilitate faster adaptation and implementation of CH technologies. The ENJECT Cooperation in Science and Technology (COST) action [16] initiative to summarize the existing readiness of European countries to adopt CH included an investigation into CH education curricula and revealed disappointingly modest efforts toward CH throughout third-level education [10]. The survey presents responses across 15 European countries to questions that assessed the prevalence of university-based programs that educate and equip health care professionals or future health care professionals to engage with CH. The results showed a reliance on biomedical informatics courses, with less than half of the sample programs offered by respondents having a CH or electronic health dimension. More than half (22 out of 42) of the programs cited had health care informatics as a major component. The majority of these 22 programs included the phrase *health informatics* or *information management* in their titles. This snapshot of health professional education indicated a lack of progress in the incorporation of CH into their program content, leading us to question the broader interdisciplinary context around CH education. If health care professional educators were struggling to span the CH interdisciplinary divide, then how might other relevant disciplines be faring in this regard? Given the inherently interdisciplinary nature of CH, we set out to qualitatively develop deeper understandings of the challenges that might underpin the ENJECT findings. Although core elements of an interdisciplinary program at the fourth level (PhD) have been proposed based on the experience of Marie Curie Actions for funding early-stage researcher programs [17], such context-specific methods for interdisciplinary education (IDE) cannot simply be transferred to all levels and contexts of academic education without more research and adaptation. This paper addresses that deficit by exploring how third-level IDE in the domain of CH might be leveraged to secure future development and practice of CH. Important questions concerning CH education are raised in relation to the disciplines involved, professional boundaries, crossing boundaries, and the optimum ways in which to deliver interdisciplinary CH education.

With some notable exceptions, previously presented, existing literature offers few guidelines as to the design of IDE modules for CH teams, no advice on how much interprofessional education (IPE) or IDE is adequate, and no indication of how its objectives should be defined. The aim of this study was to examine the perceptions of researchers engaged in interdisciplinary research regarding the design and development of CH education modules.

### Related Work

#### Interprofessional Versus Interdisciplinary Education: Fundamental Concepts

IPE and multiprofessional education are examined in the literature [18]. There is some discrepancy in the literature regarding the terminology used to describe bringing various disciplines or professions together to learn. IPE is defined as occasions when students of two or more professions learn with, from, and about each other to improve collaboration and the quality of care and services, according to the Centre for the Advancement of Interprofessional Education [19]. IPE is well established in health care education. The learning objectives, as expressed in the study by L’Ecuyer [20], are (1) to communicate the professional roles and responsibilities of all team members clearly to others, (2) to understand the relationship between effective team communication and improved patient safety and health outcomes, and (3) to demonstrate skills of effective interprofessional team and patient-centered communications that integrate the knowledge and experience of other health professionals and patients to provide appropriate care. Although well established in the health professional sphere, IPE does not often encompass key nonclinical members of the contemporary health care team, such as medical engineers and informaticians or medical data analysts. Although the term has traditionally been defined solely in the context of health care professions, we propose that IPE now needs to be seen in a broader context. If CH research is to achieve its full potential, then there is a need for IPE to embrace professionals beyond health care—in particular engineers, computer scientists, physicists, and mathematicians who have a crucial role in designing the technologies required for CH. IDE, on the other hand, is defined as [21]:

An interaction involving collaborations between students from differing subject areas in pooling their disciplinary knowledge in addressing complex and significant, real-world problems [leading to] the ability to understand and be understood by a diverse group of specialists.

Smith and Clouder [22] explored the similarities and differences between interprofessional and IDE. As mentioned, definitions of interprofessional learning tend to link more directly to practice and the workplace than definitions of interdisciplinary learning. However, both explanations stress the centrality of collaboration and integration toward addressing complex problems. Emphasizing the synthetic procedure in the study of Aboelela et al [23], interdisciplinary research integrates the analytical strengths of two or more often disparate scientific disciplines to solve a given biological problem. While engaging in this mission, the terminologies, approaches, and methodologies may be gradually merged, and the scope of investigation may broaden and may even lead to new hybrid disciplines.

Overall, within an interdisciplinary team, *each team member builds on each other’s expertise to achieve common, shared goals, for example, toward integrated care.* In this paper, between IPE and IDE, the latter term is adopted.

#### Challenges, Barriers, and Descriptors of Interdisciplinary Work

Indicatively, IPE barriers have been identified in a study by Hall [24]: each health care profession has a different culture, which includes values, beliefs, attitudes, customs, and behaviors that contribute to the challenges of effective interprofessional teamwork. In this work, a clear and recognizable idea or goal, serving as the focus for team members, is suggested as an opportunity for addressing such barriers for teamwork to succeed. This *idea dominance* allows each member to shift from their specific professional focus to one requiring an understanding of another’s observations and interpretations. Problem-based learning (PBL) for IDE and IPE can support this idea and can be a vector of success in the context of CH.

Beyond IPE among health professionals, CH training involving all relevant disciplines is not well explored. Attitudes toward interdisciplinary training have been studied where staff and students have been drawn from medical and engineering backgrounds. In a study by Spoelstra et al [25], medical and engineering students and staff attitudes were examined, and important differences were reported for staff and students between the disciplines regarding attitudes toward and perceptions of the relevance of interdisciplinary learning opportunities and the role of creativity and innovation. There was agreement across groups concerning preferred learning, instructional styles, and module content. Medical students showed greater resistance to the use of structured creativity tools and interdisciplinary teams. Such attitudes could be dealt with early in an educational program. As mentioned in the study by Feyerabend [26], viewing science too ideologically and rigidly, similar to a religion, and becoming dogmatic impair the overall progress of science.

Mountford et al [27] comment on the increase of interdisciplinary research networks at the doctoral research level to increase innovation, creativity, and knowledge and focus on three such CH doctoral research networks that have been funded by the EU. They raise concerns as to the structuring of these networks to accomplish both deep disciplinary goals and broader interdisciplinary objectives at the same time. On the basis of 28 semistructured interviews with the doctoral students on these programs, they outline three key elements to enhance the development of interdisciplinary social capital within such networks: structuring the program to facilitate the extraction of value for each student from the interdisciplinary process, motivating students throughout the interdisciplinary program journey, and facilitating students to relate to others both within and external to the program.

From another perspective, the value of interdisciplinarity in research has been critically examined [28]. Various researchers have explored the value of interdisciplinarity in terms of citation and funding. In a study by Bromham et al [29], it was found that the greater the degree of interdisciplinarity, the lower the probability of being funded, whereas in the study by Larivière et al [30], it was found that distance in interdisciplinarity increases scientific impact of publications.

These studies introduce descriptors of interdisciplinary research [31], having as a basis the work of Stirling [32,33]. In that work, *variety*, *balance*, *and disparity* were introduced as indicators of disciplinary diversity. *Variety* refers to the number of disciplinary categories, *balance* is related to the evenness of the distribution of disciplines, and *disparity* measures the extent to which these disciplines are different or similar from a cognitive point of view. Such descriptors may be useful in describing necessary or typical interdisciplinarity in CH education and research.

#### Paradigms of Interdisciplinary Education

IDE assumes innovative teaching methodologies, as it should foster active students’ involvement, exchange of opinions, and cooperation. The most widely used paradigms for IDE include competency-based learning, PBL, project-based learning, and design-based learning. Competency-based learning builds students’ knowledge, one competency at a time. In that sense, it is a natural way to introduce different disciplines from along the continuum of learning into the students’ body of knowledge. The formative method of assessment used in competency-based learning places an emphasis on the application of knowledge in a certain situation (problem), focusing this educational paradigm on the skills acquired by the students [34]. As specific individual skills might be more challenging to obtain for different students, the collaborative learning approaches can be used to provide more flexible learning environment. The work done by Hall Barber et al [35] presents the results of the introduction of competency-based learning approach in medical studies. To enable students from different disciplines to work together, *PBL* has been used in IPE for health care professional students [36]. It has been considered as a means of encouraging self-directed learning, critical thinking, lifelong learning, and self-evolution among students. *Project-based learning* [37] involves the solution of a problem set by the student or instructor. This question or problem in focus serves to organize and drive activities toward a *solution that addresses the driving question*. It involves initiative by the student or group of students, and a variety of educational activities constitute parts. It usually results in a product (eg, a report and a computer program) delivered after a considerable length of time and investment of work effort. Teaching staff only play a facilitatory role in the learning process.

When considering project-based learning versus PBL, the starting point in both approaches is a problem; however, in PBL, students’ activity is directed to *studying*, whereas in project-based learning, students’ activity is directed to constructing the solution or product [37].

Design-based learning has recently been proposed [38] as a means to help to bridge the gap between research and practice in medical education because it contributes toward both theory testing and refinement on the one hand and improvement of educational practice on the other hand. This genuinely introduces interdisciplinarity [39]. Its main aspects are (1) iterative cycles of design, evaluation, and redesign; (2) authentic real-life learning settings; (3) testing and refining theories as well as advancing practice; (4) mixed methods studies; and (5) interaction among designers, researchers, and practitioners with different expertise.

Project-based learning and design-based learning methods seem to present differences, as discussed in the study by Stokholm [40], not only in the procedure of learning but also in the foreseen competence creation. The former leans more toward discursive thinking and an analytical-oriented working mode, whereas the latter toward design and innovation theories, methods, and tools as well as a culture of systemic thinking and a synthetic-oriented working mode.

## Methods

### Overall Methodology

To address the aim of this work and explore academic IDE toward the evolution of CH, a cross-institutional (Aristotle University of Thessaloniki [AUTH], Greece, and University College Dublin [UCD], Ireland), multistep qualitative approach was defined. Although these institutions are geographically distant, they are similar in many respects. Both universities are state funded, and they both have highly competitive, long-standing, and sought-after professional health education programs (nursing and medicine) and computer science and engineering programs. They also have well-developed collaborative interdisciplinary postgraduate research education programs, which have a CH ethos at their core. The AUTH Lab of Computing and Medical Informatics and Biomedical Imaging Technologies (MI-LAB) and the UCD Insight Centre for Data Analytics are international leaders in CH research and have collaborated in several EU-funded CH projects, for example, the initial training network “Connected Health Early Stage Researcher Support System” and the project “Championing a Multi-Sectoral Education and Learning Experience to Open New Pathways for Doctoral Students.”

The data collection was conducted in two stages:

- Stage 1: interviews with CH researchers to explore specific themes
- Stage 2: workshops to further examine and elaborate on themes emerging from stage 1

Stage 1 research was undertaken in AUTH, whereas stage 2 data collection was undertaken in UCD.

### Stage 1

Interviews were undertaken with researchers in the health informatics sector collaborating with the AUTH MI-LAB, Greece. The purpose of the interviews was to gain insight regarding the views of researchers about the needs for CH education and the ways in which CH education could be best delivered. Ethical review was not required as this study was the evaluation of standard educational practices. All participants consented to participate and were assured of confidentiality and anonymity. The interviews were undertaken by an experienced qualitative researcher who has published several papers that have employed similar research methodologies [27,41-43].

All staff and postdoctoral and doctoral researchers employed in or closely collaborating with AUTH MI-LAB in health informatics research or education were invited to participate in the study. A total of 9 individuals agreed to be interviewed, comprising 2 staff and 7 early-stage researchers (at the postdoctoral or PhD level). The majority of those interviewed were engineers or computer scientists, who had specialized in health informatics. All those interviewed had undertaken their research in an interdisciplinary context and had experience of communicating with a broad range of disciplines. All researchers had contributed to the health informatics module delivered to the undergraduate medical students at Aristotle University. The interview schedule is presented in Multimedia Appendix 1. All interviews were audio recorded, transcribed verbatim, and examined for emergent themes according to the method described by Braun and Clarke [44]*.* The technique of thematic analysis, as described in the study by Braun and Clarke, was used to analyze the interview data. Thematic analysis enables the identification, analysis, and reporting of patterns or themes that occur within a qualitative dataset while also offering a robust method of organizing, describing, exploring, and analyzing the data [45]. The interview transcripts were initially examined through the process of reading and rereading (TC and NM). The transcripts were read independently to enable the researchers to become familiar with the data. Each person then made individual observations in relation to the interview content while developing preliminary inductive codes. Working together, the authors developed a coding framework, which was subsequently used to examine the data. To ensure consistency, the authors (NM and TC) discussed agreements and disagreements to reach a consensus regarding the emergent themes. The themes were aligned with the codes, thereby enabling the development of a narrative. Through the analysis process, the perceptions regarding how interdisciplinary could be designed and delivered were examined. Qualitative data analysis software was not used in this study.

### Stage 2

This stage included workshops to gain further knowledge and insight. These workshops took place at the Insight Centre for Data Analytics, UCD, Ireland. The method used in both workshops was based on the structured group feedback approach [46]. This method was selected to garner participant responses in relation to the themes that emerged in stage 1. In all cases, the participants were informed and consented to the use of their data for research purposes, and no personal data were collected.

Workshop 1 was entitled “Finding our way for interprofessional connected health education” and followed a 3-phase procedure. In phase 1, participants were split into *education* and *teamwork* groups. Those participating in the education group had to individually reflect on four questions and answer in written form: regarding interdisciplinary educational examples; the education level for a CH course; benefits, challenges, and objectives for a CH course in an interprofessional class; and potential project setups for a CH class. Similarly, the CH teamwork group had to reflect on and write answers on interdisciplinary teamwork examples, potential concept and terminology or language barriers in interdisciplinary work, interdisciplinary teamwork barriers in CH research, and potential beliefs and attitudes to be addressed in CH teamwork. The questions are listed in Multimedia Appendix 2. In phase 2, the group members came together to discuss their answers and reach a fusion of ideas as well as a consensus or a ranking and prioritization within group. In phase 3, a plenary discussion took place. Each group rapporteur presented their results to the whole group of participants, and a final consensus was reached.

This workshop had 10 participants, all of them researchers in CH, including postdoctoral researchers (n=3), PhD students (n=4), and professors (n=3), from medicine, physiotherapy, business, social sciences, and ICT backgrounds. The duration of the workshop was 2 hours.

Workshop 2 took place during a full group meeting of CH researchers at the Insight Centre and included 16 participants, including postdoctoral researchers (n=4), PhD students (n=10), and professors (n=2). The disciplines represented were the same as those of the first workshop. The duration of this second workshop was less than 2 hours. Building on the outcomes of workshop 1, the purpose of this workshop was to refine and elaborate on specific points. Therefore, the previous procedure was followed, but with the addition of two new questions that focused on preconceptions in an interdisciplinary team that could be addressed through education and the focus of an interdisciplinary CH course (see Multimedia Appendix 3), and again, the participants were split in two groups that answered and discussed the two questions before coming back to a full group discussion.

## Results

### General Findings

The analysis of the interviews revealed a number of key themes as follows: challenges concerning IDE, recommendations in relation to developing IDE, and positioning of IDE within a curriculum (Table 1). Quotes were chosen that were illustrative of the subthemes and themes emerging, with due regard to representation across institutions, disciplines, and career stages. The interviews were complemented by the workshop results on CH education and interdisciplinary teamworking.

**Table 1:** Emergent themes and subthemes from the interviews undertaken at the Lab of Computing, Medical Informatics and Biomedical Imaging Technologies, Aristotle University.

Theme	Subthemes
Challenges identified to interdisciplinary learning	Communication, state-of-the-art knowledge, and resistance to interdisciplinarity
Recommendations for an interdisciplinary learning environment	Learning environment and link to real-world practice
Position of the interdisciplinary module within the study program	Need for skills and experience and early integration

### Interview Results From the Lab of Computing and Medical Informatics and Biomedical Imaging Technologies, Aristotle University

#### Challenges Identified to Interdisciplinary Learning

The researchers interviewed in health informatics domain at the Aristotle University identified several key themes in relation to CH and CH education. They identified several challenges that centered on working across and between disciplines. The challenges were frequently associated with communication and the difficulty experienced in communicating their own key disciplinary concepts while also understanding those of an individual from another discipline in an interdisciplinary context. They identified that each discipline perceives ideas and concepts differently and that it can take time to convey these concepts to an individual beyond their own discipline. One researcher commented as follows:

Difficult because we cannot communicate properly our needs both of us...I ask something my own words because I have something on my mind and I say because it is a different way of thinking so we have to sit down and discuss for a long time so that we can understand each other.

AUTH 2

The fast pace of technological advancement is beyond doubt; however, some concern was expressed regarding the up-to-date understanding of members of other professions within the team. One participant commented:

The problem is with the older doctors who think technology is not for them and try to put the burden on you...they too must understand things so that they can help me and their selves.

AUTH 6

The interview participants frequently referred to their links with health care staff, their need to work together, and the importance of being able to communicate and collaborate as being an essential element of their work:

...constantly in communication with the health care professionals in order to explain to them how to explain to the patients how to use our devices and our applications.

AUTH 5

A number of participants raised the possibility of resistance from other disciplines to engage in an interdisciplinary context:

I don’t know the level of willing that’s let’s just say from different departments to join forces.

AUTH 1

#### Recommendations for an Interdisciplinary Learning Environment

The staff who were interviewed had considerable experience in terms of education delivery, many of whom had delivered lectures and supported the laboratory lessons for the undergraduate medical program and curriculum. When they were asked to describe how best they thought IDE could be delivered, several critical recommendations for learning emerged.

A number of participants recommended that students should learn to work as part of a multidisciplinary team from early in their education, as they believed this would prepare them to collaborate and communicate with disciplines beyond their own from early in their careers:

...laboratory sessions for students...team orientated...multidisciplinary projects for the students in order to learn from early on how they can collaborate and how they can speak the same language.

AUTH 1

A number of participants identified the importance of learning in an enjoyable environment because they believed that this promotes better engagement and an opportunity for students to extend their learning beyond their own disciplinary limits:

...have a little bit of fun a play…play that will turn on your imagination not for hard work…that you are doing daily…let's have fun and be out of the box you know...

AUTH 3

One person identified the value of interdisciplinary work as a means of empowering students to link research and clinical outcome:

More practical things how to link the research with the clinical outcome.

AUTH 4

Another indicated that interdisciplinary work is an opportunity to innovate in terms of education and enable the introduction of contemporary topics with a view to stimulating student interest in engagement and learning:

...cutting edge and innovations which are currently hot in the science field.

AUTH 5

#### Position of the Interdisciplinary Module Within the Study Program

The interview participants made some recommendations in terms of the position of an interdisciplinary module within a program. A number of participants commented that owing to the wide variety of career pathways that can be chosen by electrical engineers and computer scientists, many of which do not involve engagements with health care, it was thought that offering such an opportunity late in an undergraduate program or as part of a master’s degree program would be most appropriate One participant commented as follows:

...only at the last years when you have chosen your faculty...because electrical engineering is versatile...its only one tenth of what engineering can do.

AUTH 6

Another participant indicated that introducing such innovations at an early stage within the formalized education cycle might be difficult; however, it could be developed initially as an extracurricular activity:

...in order to include this seminars this lesson in the everyday curriculum of high school or the first semester of college or university I think this is a little bit difficult all this could be in extracurricular.

AUTH 5

### Workshop Results From the Insight Centre for Data Analytics, University College Dublin

With respect to existing or potential cases of IDE, quite a few education examples emerged—“Patient care in the long term” or a “CH design project” (similar to a weight management program design). These cases were distinguished from other more clearly multiprofessional education examples such as biotechnology programs, patient care in *case* discussions within medical training, and training in rehabilitation programs. In these multiprofessional education examples, the role of multiple health professionals appears, but rather as forming parallel and not fusing or interacting paths. Overall, it was more challenging to find interprofessional courses.

With respect to positioning of the module within the program, two levels were identified for CH education with two different aims: (1) at undergraduate level for awareness-raising purposes, not necessarily focusing on interdisciplinarity, and (2) at postgraduate level for actual interdisciplinary research. An alternative idea was to embed elements within different courses at the undergraduate level. At the undergraduate level, it was found essential to inform students about the roles of different actors and to address misconceptions, perhaps by presenting examples of how interprofessional projects worked. To build a common language within an interdisciplinary PBL team, participants believed that some time has to be invested, and each member has to have at least an undergraduate level of proficiency at their discipline. This indicates the master’s level as an appropriate time for achieving the second aim of CH education. At later stages, it was felt that the lack of time, professional duties, and professional bias might hinder the potential involvement in interdisciplinary research teams.

Project-based and problem-based courses were discussed as a means to involve young researchers, with *fresh eyes* in interdisciplinary experience. Course setup examples were mentioned. For project-based learning, such course examples included health and well-being solutions or CH projects, whereas PBL examples included patient care at large as a topic and a hackathon as the instrument. Learning should take place based on real-life scenarios and solutions or actual clinical studies and authentic problems that have not been accommodated under traditional care scenarios. Participants suggested learning in a practical way, using real products where clinicians can see outcomes and by locating real problem-based situations and investigating how technology can solve these problems. Participants were adamant that the PBL experience should include learning to map the problem, as designers do.

In general, teamwork learning was placed as the central concept toward better understanding the domain and the team and meeting patient care needs. Learning from experience is important; therefore, an idea offered was to *swap hats* to reflect on and understand roles, for example, by encouraging the engineer to take the patient’s history and the clinician to be aware of the evolution of technology. With regard to teamwork research, a series of interesting ideas emerged. Discovering and highlighting the value of participating and working in an interprofessional or interdisciplinary team was identified as an essential process. To alleviate barriers, it is crucial to build interprofessional or interdisciplinary empathy skills as well as to understand and communicate one’s own skills to strengthen the team. Each discipline may have a different view (engineers, computer scientists, and medical doctors); therefore, an iterative procedure is needed. Participants emphasized the importance of remaining open to new ideas, understanding the gaps, and highlighting both success and failure stories. The teamwork qualities mentioned most frequently were curiosity, attitude, confidence within the team, and acceptance of complementary personality types.

## Discussion

### Overview

**Figure 1:**
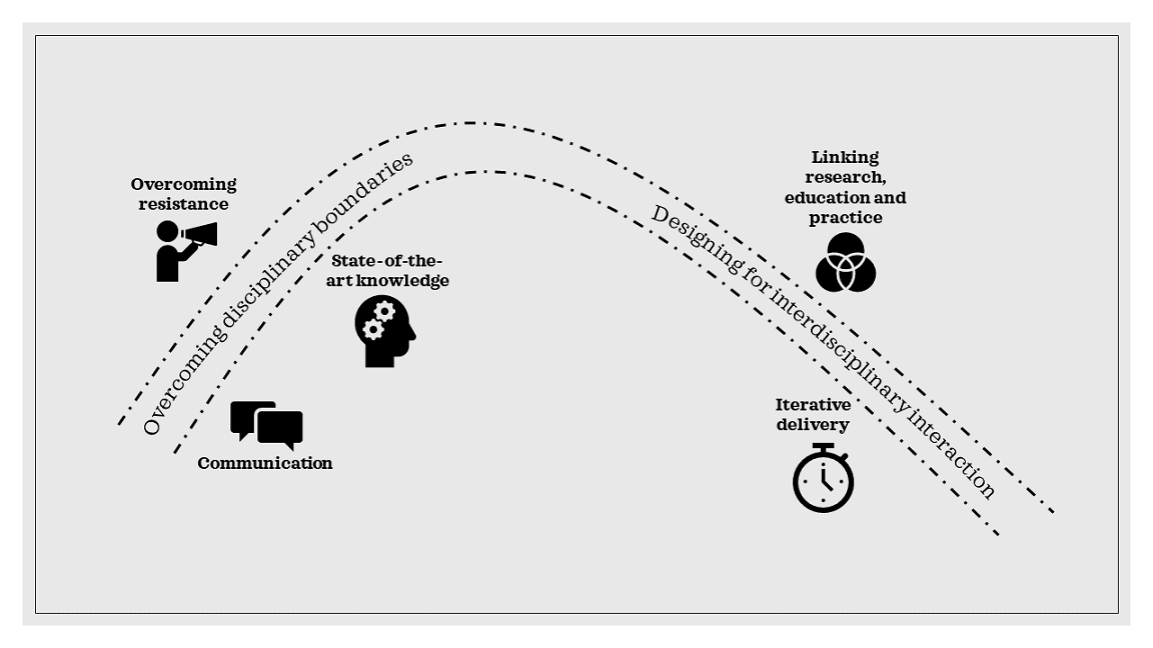
A model for interdisciplinary education for connected health.

Our research suggests two areas where improvements can be made in the synthesis and design of new CH education concepts: (1) overcoming disciplinary boundaries and (2) designing for disciplinary interaction. These build into a model for IDE for CH as illustrated in Figure 1.

### Overcoming Disciplinary Boundaries

Our research identifies three challenges that must be overcome in the design of new CH education programs to ensure cross-disciplinary engagement: communication challenges, state-of-the-art knowledge, and resistance to interdisciplinarity. We have combined insights from both the literature and the data gathered to develop suggested responses to the challenges identified as outlined below.

#### Challenge 1: Communication

In the first instance, our data indicated that difficulties regarding the different perceptions of ideas and concepts were hindering interdisciplinary endeavors. Participants also highlighted the difficulties posed by multiple disciplinary *jargons*.

#### Proposed Solution

On the basis of participants’ insistence that understanding alternate perspectives is key, we borrow from design thinking literature to suggest the use of empathy interviews to gain an understanding of multiple viewpoints. Sarasvarthy [47] presents it in the context of entrepreneurial thinking, “the point of exploring contrasting perspectives...is not to prove one superior to the other, but to learn to understand and use both”. Addressing the disciplinary language difficulty, we combine interview and workshop data to suggest the investment of time at the master’s level to develop a common language directed at CH activities that span disciplinary boundaries.

#### Challenge 2: State-of-the-Art Knowledge

The second major challenge raised throughout our research was the divergence in knowledge across disciplinary boundaries as to the availability of state-of-the-art technologies.

#### Proposed Solution

We accept participants’ recommendations from our workshops that students be encouraged to *swap hats* throughout their training to develop an understanding of the different roles of different disciplines. This may increase understanding on all sides of the disciplinary divide that their interdisciplinary colleagues are only like to be at the cutting edge of their own discipline. Just as computer scientists are unaware of the latest medical techniques, clinicians are unlikely to know of the latest software developments. This will, in turn, lead to patience and a willingness to invest time to bring interdisciplinary colleagues up to speed.

#### Challenge 3: Resistance to Interdisciplinarity

Our data indicate an unwillingness among some students, researchers, and practitioners to reach across the disciplinary divide.

#### Proposed Solution

We recommend iterative processes in the education modules as per the principles of design-based learning discussed in the Introduction section [39,40]. Social constructivist learning theory, whereby learners work together, sharing their learning and constructing a new understanding for themselves through their experiences, underpins the concept of design-based learning [48]. Our data indicate that this is required to ensure that different views are accommodated and that interdisciplinary skeptics are given time and repeated opportunities to come on board.

### Designing for Disciplinary Interaction

Our research indicates two major factors that lead to the successful design of interdisciplinary CH education: (1) the nature of the education module and (2) the positioning of the module within the education program.

#### The Nature of the Education Module

Overall, two key issues arose as to the nature of the education experience when seeking to integrate disciplines that may assist in the design of successful CH education going forward. The first is the creation of an enjoyable and challenging educational environment. Our data suggest that innovation and interest are key to the design of such an environment. The second is the necessity to link research and education together with its corresponding outcome, as our study confirms that education recipients wish to map their educational and research activities onto real-world practice. We recommend the use of both problem and project-based learning approaches to address both environment and application needs. Our workshop results suggest that although project-based activities are more suited to health- and well-being–focused challenges and educational outcomes, problem-based learning is more likely to address those that focus on patient care.

#### The Positioning of the Module Within the Education Program

Our data suggested a divergence of opinion as to whether these modules and the learning associated should be delivered early or late in the educational life cycle. Arguments for early delivery center on the advantages of early integration and the embedding of interdisciplinary attitudes. Those who advocated later delivery were concerned that the lack of developed skills and knowledge at that point might hinder useful interdisciplinarity. In response to both sets of concerns, we recommend a dual approach suggested by our workshop participants, which sees early awareness-raising activities (at the undergraduate level) followed by later problem- and project-based learning activities (at the master’s level). Introducing interdisciplinary CH learning in this way is supported by Schön’s work in relation to the role of reflection in professional learning discussed by Atkins and Murphy [49], who maintain that reflective professional learning occurs in three stages: first, creating awareness and feelings of discomfort; second, critically analyzing knowledge and feelings; and finally, developing a new perspective. In a similar vein, the spiral curriculum [50], an education design whereby topics are visited and revisited at increasing levels of complexity, allows the development of deep learning. Theory, therefore, supports our participants’ suggestion of an early introduction coupled with a later development of learning.

### Conclusions and Future Work

The CH ecosystem sets the basis for the investigation and deployment of new care models, leveraged by technology [51,52]. CH offers new opportunities for redesign and improvement of health and care, but its implementation and acceptance necessitate reorganization at multiple levels. IDE in CH is the cornerstone for broader adoption and impact of the CH paradigm and a prerequisite for research advancements in the CH field. However, such educational activities are not widely developed [11], serving as motivation for this study that focuses on identification and understanding of barriers and challenges in CH IDE.

This study relied on surveyed opinions and views across multiple disciplines, attempting to provide some insights on the existing challenges and to indicate potential directions for (successful) implementation of CH education. It mainly focused on the inherent interdisciplinarity in the field, explored the interdisciplinary-related barriers, and offered solutions for overcoming interdisciplinary boundaries and designing CH curriculum.

We consider this work as a first step in investigating pathways to successful CH education. Although this work might be limited in terms of number of organizations involved in the workshops used to define the themes and challenges of interdisciplinary education, the number of identified disciplines mentioned in the obtained themes and challenges reassures the quality of achieved results. A wider multicountry mapping of needs and ideas would further contribute in terms of context-related barriers and enablers as well as in the formation of multicultural CH educational networks. To create solid evidence in this area, the work of Car [53], which proposes a methodology for systematic reviews in digital health education, can be expanded in this direction, encompassing the interdisciplinary aspects of CH education. In addition, recent evidence that new digital education tools such as virtual reality can improve knowledge and skills of health professionals [54] indicates that the challenges of interdisciplinary CH educations could be further explored via virtual reality and similar innovative means.

This work contributes useful inputs for CH curricula design with a focus on interdisciplinarity, both with regard to the alleviation of barriers and the design of interaction between different stakeholders in CH.

## Appendix

Multimedia Appendix 1 Interview schedule.

Multimedia Appendix 2 Workshop 1 questions.

Multimedia Appendix 3 Workshop 2 questions.

## Abbreviations

AUTH: Aristotle University of Thessaloniki
CH: connected health
COST: Cooperation in Science and Technology
ENJECT: European Network for the Joint Evaluation of Connected Health Technologies
EU: European Union
ICT: information and communications technology
IDE: interdisciplinary education
IPE: interprofessional education
mHealth: mobile health
MI-LAB: Lab of Computing and Medical Informatics and Biomedical Imaging Technologies
PBL: problem-based learning
UCD: University College Dublin
