# Supplementary material for: Leveraging Interdisciplinary Education Toward Securing the Future of Connected Health Research in Europe: Qualitative Study
Source: J Med Internet Res. 2019 Nov 13;21(11):e14020. doi: 10.2196/14020 (PMC6881783; doi:10.2196/14020)
Supplement: Multimedia Appendix 1 [file jmir_v21i11e14020_app1.pdf]

Appendix 1. Interview Schedule

1. Talk to me about you own background in terms of education.

2. Explain to me a little about your own research.

3. Tell me about the links between your own work and the delivery of healthcare.

4. Do you undertake research with other disciplines (tell me a little about that, how do you find it, how did you develop a common understanding?).

5. Do you deliver education to other disciplines how do you find that; what format does that take?

6. We would like to see students across disciplines involved in “connected health” working together more closely at an earlier stage in their education…..do you have some suggestions as to how this could be achieved?

7. What do you think might be the barriers?

8. What do you think might be the facilitators for this?

9. What do you think the module should cover in terms of content, topics?

10. Do you have any suggestions as to how it might be delivered?

11. Interprofessional education is considered to be when 2 or more professions learn from with an about one another to improve collaboration and quality of care…..do you think this approaches to CH education could fit under this term or do we need a new name for this approach to educating the next generation of CH professionals?
