# Supplementary material for: Leveraging Interdisciplinary Education Toward Securing the Future of Connected Health Research in Europe: Qualitative Study
Source: J Med Internet Res. 2019 Nov 13;21(11):e14020. doi: 10.2196/14020 (PMC6881783; doi:10.2196/14020)
Supplement: Multimedia Appendix 2 [file jmir_v21i11e14020_app2.pdf]

Appendix 2. Workshop 1 questions

Interdisciplinary connected health teamwork

Q1. Interdisciplinary: A knowledge view and curriculum approach that consciously applies methodology and language from more than one discipline to examine a central theme, issue, problem, topic, or experience.

Multidisciplinary: The juxtaposition of several disciplines focused on one problem with no direct attempt to integrate.

Can you think of an interdisciplinary vs multidisciplinary team-work example?

Q2. From your experience can you mention a concept that discipline X and discipline Y use in a totally different manner?

Have you ever tried to explain a concept to the other part in such a case?

Do you have a suggestion how to develop a common language? Do you see a role in education there?

Q3. Do you see any Interprofessional teamwork barriers in a Connected Health research and development team?

Q4. Do you think there are beliefs and attitudes about other professions involved in CH teamwork that need to be alleviated? Do you see a role in education for that?

Interdisciplinary connected health education

Q5. Interdisciplinary: A knowledge view and curriculum approach that consciously applies methodology and language from more than one discipline to examine a central theme, issue, problem, topic, or experience.

Multidisciplinary: The juxtaposition of several disciplines focused on one problem with no direct attempt to integrate.

Can you think of an interdisciplinary vs multidisciplinary educational example?

Q6. When should a CH course be taught? (Undergraduate level? Graduate?)

Q7. Do you see any benefit in a Connected Health course being taught in an interprofessional class?

If Q7 yes

Q7.1 Should an CH course blend independent discipline learning and IP teamwork

Q7.2. Would you envisage a term for CH IP course

Q7.3. Would the following be objectives of a CH IPE course

- Communicate your professional role and responsibilities to other professionals
- Explore how you will work together as a team to meet patient care needs
- Understand how to effectively communicate and interact to enhance team function
- Identifying the key challenges associated with embracing Connected Health technologies.

Q7.A If you agree with the objectives, do you have any ideas how to implement them?

If Q7 no

Q7.4 An IP CH course would mostly fall in a *Potpourri Problem?*

Q7.5 An IP CH course would mostly fall in a *Polarity Problem among professions*

Q7.B If yes in Q7.4 or Q7.5, can we do something to avoid such problems?

Q8. Can you give an example of a project-based learning or a problem-based learning setup for a CH course? Which one (project or problem) would you choose and why?

It was explicitly mentioned that '*the papers will be collected for further analysis, so make sure you are comfortable with what is written in the paper you deliver.'*
