# Supplementary material for: Leveraging Interdisciplinary Education Toward Securing the Future of Connected Health Research in Europe: Qualitative Study
Source: J Med Internet Res. 2019 Nov 13;21(11):e14020. doi: 10.2196/14020 (PMC6881783; doi:10.2196/14020)
Supplement: Multimedia Appendix 3 [file jmir_v21i11e14020_app3.pdf]

Appendix 3. Workshop 2 questions.

Q1. In the context of collaboration in CH we now know from our work to date that people have preconceptions e.g. “traditions”, “are clinicians ready for this”, “it is working fine, why change it”, hierarchy, “some disciplines are seen as more important”, etc. How would you propose we address these in terms of developing an education course?

Q2. If we were to setup an interdisciplinary CH course, what are the important areas we should cover, what topics should be included? What should its focus be?
